# Formation of neocortical memory circuits for unattended written word forms: neuromagnetic evidence

**DOI:** 10.1038/s41598-018-34029-y

**Published:** 2018-10-25

**Authors:** Eino J. Partanen, Alina Leminen, Clare Cook, Yury Shtyrov

**Affiliations:** 1 Center of Functionally Integrative Neuroscience (CFIN), Department of Clinical Medicine, Aarhus University Hospital, Denmark (Aarhus University Hospital, Nørrebrogade 44, Building 10G, 5th floor, 8000 Aarhus C, Denmark; 2 Cognitive Brain Research Unit, Department of Psychology and Logopedics, Faculty of Medicine, University of Helsinki, Helsinki, Finland (PO Box 9 (Haartmaninkatu 3), FI-00014 Helsinki, Finland; 3 St. Petersburg State University, St. Petersburg, Russian Federation; 4 MRC Cognition and Brain Sciences Unit, University of Cambridge, 15 Chaucer Road, Cambridge, CB2 7EF England

## Abstract

To master linguistic communication, humans must acquire large vocabularies quickly and effortlessly. Efficient word learning might be facilitated by the ability to rapidly acquire novel word forms even outside the focus of attention, occurring within minutes of repetitive exposure and suggesting fast and automatic lexicon acquisition. However, this phenomenon has been studied in the auditory modality only, and it is unknown whether similar mechanisms also exist in the visual domain. We tested this by presenting participants with novel written word forms while the focus of their attention was on a non-linguistic dual colour-detection task. Matched familiar word forms served as a control. Using magnetoencephalography (MEG), we scrutinised changes in neuromagnetic responses to familiar and to novel word forms over approximately 15 minutes of exposure. We found, for the first time, a visual analogue of automatic rapid build-up of neural memory circuits for unattended novel lexical items, seen as a rapid enhancement of early (~100 ms post-onset) activation in the left anterior-superior temporal lobe. Our results suggest that the brain quickly forms cortical representations for new written forms, and indicate that the automatic neural mechanisms subserving rapid online acquisition of novel linguistic information might be shared by both auditory and visual modalities.

## Introduction

Acquisition of large vocabularies is a prerequisite for language use and efficient linguistic communication. In children, word learning is extremely rapid and new word forms can be learned and accurately used after a handful of repetitions (e.g.^1^), with some studies assessing the learning rate to be as high as 10–20 words per week (e.g.^2^). While slower in adults, word learning continues to take place throughout lifespan; behavioural studies suggest that adults can start using a new word in semantic tasks already after five 20-minute daily training sessions^3^.

As, behaviourally, humans are capable of acquiring at least some knowledge of a word after only few repetitions, it is plausible that the *neural processes* underlying formation of memory traces for new lexemes in the brain are extremely rapid. However, little is known about the brain underpinnings of language learning and word acquisition. The acquisition of a novel surface word form per se, on the one hand, and of its semantics, on the other one, may be underpinned by different mechanisms and could therefore be dissociated. For instance, mere exposure to novel spoken patterns (i.e., word forms without a specific meaning) has been shown to lead to a new memory trace formation in the mental lexicon^4^, which could be the first step, or even a pre-requisite, in a (possibly slower) neural process towards a fully-fledged lexico-semantic word representation in the brain^5^. Previous studies have also suggested that this surface-form acquisition could be a primitive learning mechanism, especially important in early stages of language acquisition^6^. Some recent electrophysiological (EEG) studies addressed acquisition of spoken word forms online, during a single experimental session. They have shown that new neural memory traces for novel spoken word forms with native phonology (so called pseudo-words as they follow phonological and phonotactic rules of a language but have no meaning) could be formed during mere 15–30 minutes of passive exposure to them (e.g.^7–10^).

Most of these experiments have utilised the so-called passive (i.e., unattended) oddball paradigms where the novel word form (pseudo-word) is presented sparsely among frequently presented acoustically similar filler stimuli^7,8,11^. The unattended oddball paradigm has been argued to be a sensitive tool for registering transient activity related to long-term memory trace activation in the brain, reflected in an early (with latencies of ~50–150 ms after the recognition point) enhanced electrophysiological response for familiar language sounds that exceeds the amplitude of the event-related potential or field (ERP, ERF) elicited by similar but unknown (or less well known) sounds^12–14^. This enhancement is argued to stem from strongly connected neuronal assemblies, acting as word memory circuits, which form as a product of Hebbian-type learning and are robust enough to activate quickly and automatically when the respective stimulus is present at the input^15^. Furthermore, these early word-elicited activations exhibit category-specific sensitivity to the stimulus semantics, such as action ref.^16^, grammatical class^17^ or emotional content^18^.

Previous studies have shown clear linkage between changes in brain response magnitude and behavioural learning outcomes^9^, as well as with individual language-acquisition experience^19^. Furthermore, this rapid functional response re-organisation related to word form acquisition seems to be an automatised process, as similar patterns of activation dynamics were found in both active listening and in passive exposure^9^. At the same time, this automatic mechanism may be specific to native language phonology (at least in adults, e.g.^20^; however, see^10^). The observed response enhancement has been proposed to reflect Hebbian synaptic strengthening resulting from correlational neuronal activity^21^, the account also supported by computational simulations^22,23^.

In terms of sources of neural activity underlying rapid word form acquisition, these previous EEG studies suggested the memory-trace related activation increase to be mostly underpinned by the enhancement of activation in the left perisylvian network, including left temporal and inferior frontal regions. However, these studies are rather sparse and have only been conducted using EEG, which lacks the spatial accuracy in pinpointing the sources of neural activity. Therefore, the use of a method with higher spatial resolution could shed light on the precise neural substrates underpinning this phenomenon. As the memory-trace related activations are small in amplitude and short in duration, a method that could combine both temporal and spatial resolution would be ideal to pinpoint the neural word acquisition processes in both space and time; thus, magnetoencephapholography (MEG) appears to be an optimal technique to further advance this research.

An even further confound in previous studies is that they have been conducted using auditory stimulation, which in itself activates parts of the perisylvian network as part of basic auditory processing. Thus, to tease apart the memory circuit build-up per se and basic sensory processing, it appears necessary to test these processes using a different sensory domain. Language is essentially a multi-modal function, relying in most individuals on at least the auditory and the visual systems. The concept of linguistic memory traces should therefore include representations of both spoken and written forms, and it thus stands to reason that similar automatic memory-trace build-up mechanisms might operate for word forms in *the visual (written) domain*. This, however, has not been tested so far.

Thus, the current experiment was constructed to address these open questions. To assess non-attentive aspects of word form acquisition, we presented the participants with novel word forms outside the focus of attention, while occupying their attention with a demanding non-linguistic task. By using an attention-demanding primary task, we could control that the participants were not intentionally focusing on the linguistic stimuli of interest. By presenting these novel word forms visually we could focus on neural dynamics related to the new linguistic stimuli, while at the same time avoiding purely auditory activation of perisylvian areas. As the present study was the first of this kind in visual modality, we opted to utilise a paradigm closely following the previous auditory research on the subject, in order to obtain comparable results. Thus, we applied a non-attend oddball paradigm, an established tool for probing memory traces for word forms in the brain, but adapted this paradigm to the visual modality of presentation. We assessed both event-related responses in signal space and cortical generators of neural activity underlying surface neurophysiological dynamics by utilising high-resolution MEG recordings and distributed source reconstruction techniques. In keeping with previous findings, we focused on the early memory-trace related activity at ~100 ms. We expected, as shown by previous studies conducted in the auditory modality^12–14^, enhancement of the early event-related responses for the novel word forms in the left perisylvian language network over the course of the experiment. As control stimuli, we used highly similar known words, for which we did not predict a similar enhancement dynamics; instead, these well-formed memory circuits may be expected to show a stable response amplitude, or even its decline due to repetition-related habituation. However, as there most likely is no perfect match between early responses to stimuli presented in auditory and visual domains, we analysed the later activity ad hoc for changes in aforementioned dynamics as well.

## Methods

### Participants

Seventeen healthy adult native English-speaking university student volunteers (age 20–40, mean = 29.76) gave their informed consent to participate in the experiment. All participants had either normal or corrected-to-normal vision. None reported any neurological, developmental, cognitive or language deficits or substance abuse. The study was approved by the local Research Ethics Committee (Cambridge, UK) and conducted in accordance with the Helsinki Declaration. Participants gave written consent and were paid for their participation.

### Stimuli

**Figure 1:**
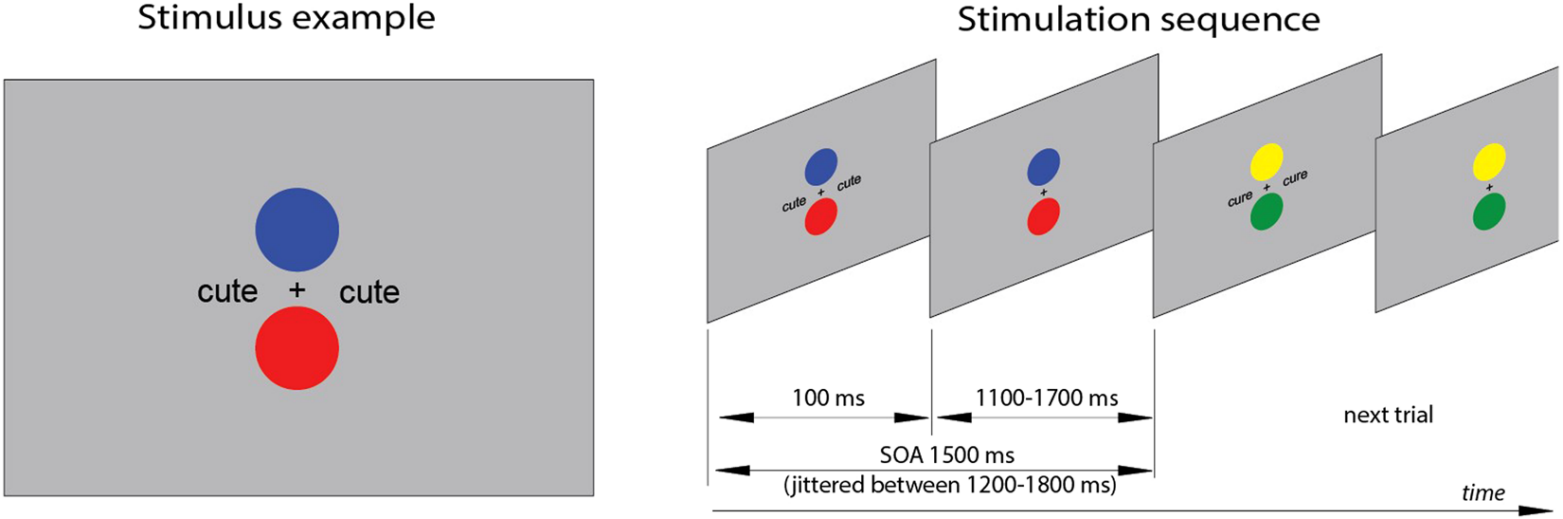
Experimental procedure. The subjects had to assess the colour of both the upper and the lower circles and respond only when a particular combination of colours/locations were present (i.e., when the task was to detect e.g. the “upper red, lower blue” target, responses to any other combination - including “upper blue, lower red” - were considered incorrect).

The visual stimulation paradigm was created in order to maximally resemble the previous auditory non-attend lexical oddball designs. To that end, two concurrent stimulation streams were visually delivered: an unattended lexical oddball stream and an actively attended non-linguistic task. The oddball stimuli, consisting of familiar and novel word forms, were presented tachistoscopically for 100 ms, with stimulus onset asynchrony jittered between 1200–1800 ms (mean 1500 ms), in black font-face on grey background (see Fig. 1). The stimuli were simultaneously displayed at symmetric locations in the left and right hemifields at 1.5° angle from the centre of the screen. We opted to utilise a symmetric bilateral presentation to ensure that the participant’s gaze was not prompted to saccade from the central task to the orthographic stimuli (the risk of which could be higher were a single asymmetric presentation method utilised instead).

Four familiar English word forms (*cure*, logarithm of instance per million words frequency according to Davies, 2013: 2.30; *pure:* 2.86; *cute:* 1.99; *mute:* 0.43) served as real words. Four novel word forms were generated from the real words by changing the initial consonants (*hure, mure, pute, hute*). All the familiar and novel word forms in each subgroup (i.e., ending in both ‘ure’ and ‘ute’) were closely matched by bigram frequency in English. Their presentation as frequent or infrequent stimuli was counterbalanced for lexicality and orthography (see *Procedure* below).

As the main experimental task, the participants were instructed to concentrate on two circles of different colours continuously displayed above and below the central fixation cross (see Fig. 1). All possible combinations of red, green, blue and yellow were used. These combinations changed in synchrony with the familiar and novel word form stimuli that appeared on visual periphery. However, unlike the latter, the circles were kept on the screen for the entire duration of the SOA in order to avoid visual onset and offset responses. Thus, the circles were seen as present continuously, with only their colours changing.

### Procedure

The experiment consisted of four blocks presented in pseudo-random order, during which the subjects were instructed to fixate their gaze on the fixation cross in centre of the screen, and to focus on the dual visual task of detecting colour circle combinations. In this task, thesubjects were required to assess the colour of both the upper and the lower circles and respond by pressing a button with the left index finger only when a particular combination of colours/locations were present (i.e., when the task was to detect e.g. the “upper red, lower blue” target, responses to any other combination - including “upper blue, lower red” - were considered incorrect). Target combination probability was 4%. Different targetcombinations were used in each block, and were counterbalanced across subjects. Within each block, the sequences of visual stimuli were individually randomised. To verify that subjects focused on detecting the colour circle combinations, an MEG-compatible eye tracking device (SensoMotoric Instruments, SMI, Berlin, Germany) was used to record the distance of the gaze from the fixation cross.

While the subjects were occupied with the dual visual task, unattended familiar and novel word forms were presented for 100 ms symmetrically in the left and right hemifields (see Fig. 1). The subjects were not informed of these stimuli, nor were they encouraged to pay attention to them. The blocks were counterbalanced to ensure that familiar and novel word forms were presented both as frequent (p = 83.6%) and as infrequent (p = 16.7%) stimuli. A total of 600 stimuli were presented (500 frequent stimuli, 100 infrequent ones) in each block. The familiar and novel word forms were presented pseudorandomly, with at least two frequent stimuli presented between any two infrequent ones. In each of the four blocks of the experiment, different combinations of frequent and infrequent stimuli were used, ensuring an overall balanced presentation: *cute* vs. *cure* (familiar word form frequent vs. familiar word form infrequent), *pute-pure* (novel vs. familiar), *mute-mure* (familiar vs. novel), and *hute-hure* (novel vs. novel). In all blocks, the only difference between the frequent and the infrequent stimuli was the change in the third letter of the stimulus. Importantly, although the stimuli were highly similar, and the visual contrasts were kept identical, the critical infrequent stimuli that (unlike the repetitive frequent stimuli that serve as fillers) are known to produce robust lexical effects were either two familiar word forms (*cure, pure*) or two novel previously unencountered word forms (*hure, mure*), allowing to compare their dynamics largely unconfounded by purely visual or other extralexical factors.

### MEG recording and data analysis

MEG was recorded using a Vectorview™ whole-head MEG system (ElektaNeuromag®, Elekta Oy, Helsinki, Finland) with 1000 Hz sampling rate. Additionally, electrodes were placed at the outer canthi to monitor the horizontal EOG, above and below the left eye to monitor vertical EOG, and on the upper body to monitor the subjects’ heart rate. The head position inside the MEG device was assessed by activating four indicator coils in relation to the cardinal points of the head (nasion, left and right preaurical points), which were identified prior to the experiment using an Isotrak 3D-digitiser (Polhemus, Colchester, VT, USA). To correct for head movements and minimise the contribution of magnetic sources from outside the head and to reduce any artifacts, the data from the 306 sensors were post-processed offline using a temporal extension of the Signal Space Separation (tSSS) method (^24^, as implemented in MaxFilter software, Elekta Neuromag). Static bad channels were detected and excluded from subsequent processing steps, compensation was made for within-block head movements (as measured by HPI coils, with HPI step set to 200 ms) and externally generated artifacts were removed. For compatibility between different recordings, the data were converted to standard head position (x = 0 mm; y = 0 mm; z = 45 mm; no correction exceeded 20 mm). Data were further analysed using Brainstorm software^25^. Eye blinks were detected on the basis of the activity in the bipolar vertical EOG channel, and corrected for using signal space projection algorithm, as were artifacts arising from heartbeat. A bandpass filter of 0.01 to 45 Hz was used. Data were then divided into epochs of 1000 ms, starting from 100 ms prior to stimulus onset. All epochs were baseline corrected to prestimulus baseline from −100 to 0 ms, and all epochs where MEG activity exceeded 3000 fT/cm on any gradiometer channel were rejected from further analysis.

As infrequent stimuli have been suggested to be more sensitive to lexical stimulus properties than the frequent stimuli which are subject to repetition suppression to a higher degree^26^, only responses to infrequent stimuli were analysed further, similarly to previous studies in the auditory domain^7,9,10^. In settings where the stimuli are not in the focus of attention, they are likely not processed further and do not result in neural memory trace activation (for analysis of frequent stimuli, see Supplementary data). To assess the build-up of neural memory traces, four sub-averages were formed. Response to the novel infrequent stimuli ‘hure’ and ‘mure’ presented in the beginning of exposure (initial 1/3 of the experiment, ca.5 minutes; 39–64 accepted epochs, mean 56) were averaged together, as were response to the same items presented at the end of exposure (final 1/3 of the experiment; 37–64 trials, mean 53). In a similar fashion, the familiar infrequent stimuli ‘cure’ and ‘pure’ presented in the initial 1/3 (mean: 56, range 44–64) and the final 1/3 of the experiment (mean: 53, range 37–63) were averaged together.

**Figure 2:**
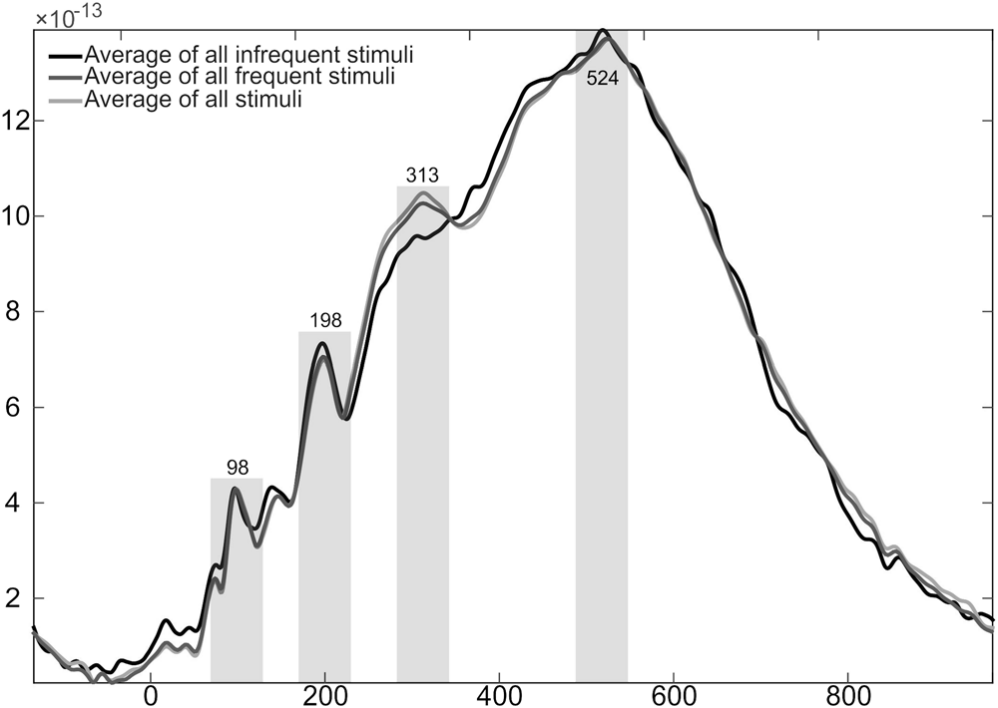
Global field power waveform of the stimuli comprising of all the accepted epochs in all conditions in the experiment. The three lines represent average of all infrequent stimuli (black), all frequent stimuli (dark grey), and average of all stimuli (light grey). Four peaks were identified from the waveform, at 98, 198, 313, and 514 ms from stimulus onset, respectively. An additional peak may exist at approximately 146 ms from stimulus onset; for analysis of this peak, see Supplementary Data.

To determine the most prominent responses in the ERF waveform in an unbiased fashion, a group-average global field power (GFP) waveform calculated based on all the accepted epochs and gradiometer pairs in the frontotemporal regions averaged across all conditions in the experiment (Fig. 2), to identify periods of maximal brain activity irrespective of the stimulus type. This approach allows for selection of periods when the brain was responding with a largest amount of activation, thus having the best SNR for follow-up analyses unconfounded by differences between conditions (thus preventing any “double-dipping” often found in ERP/ERF studies; see, e.g.^27^, using GFP analyses). Based on visual inspection of this global average activation time course, four peaks were identified: at 98 ms, 198 ms, 313 ms, and 524 ms from stimulus onset. As previous studies (see the Introduction) have shown both lexicality effects in non-attend designs and the related rapid lexical acquisition effects at latencies of ~100 ms, and it is unlikely that neural processes prior to 50 ms represent lexical acquisition effects (see, e.g.^28^; for results on the time course of neural activation to visually presented words, see^29^), we a priori focussed on the first peak. We, however, analysed the later activity ad hoc as well, as early responses to auditory and visual stimuli are not perfectly identical. As this analysis procedure resulted in multiple ANOVAs, the findings from the ad hoc analysis of the additional peaks at 198, 313 and 524 ms from stimulus onset should be cautiously interpreted. The mean ERF amplitudes in 60-ms wide windows centred at the above peak latencies were determined from each participant from a cluster of 14 channels (seven gradiometer pairs) over the left temporo-frontal perisylvian areas. The number and location of the channels were chosen in order to properly cover the temporal and inferior frontal regions, where the neural effects of rapid acquisition of novel word forms have previously been reported^7,9,19^. Furthermore, this relatively large number of channels was chosen to ensure that the result is not due to changes in in activation in one or two sensors only (e.g.^7^) but is robust enough to be seen across multiple spatially separated sensors. Although, based on the well-known language laterality, we specifically expected to find effects pertaining to formation of neural memory traces for novel word forms in the left hemisphere, a similar analysis was conducted using a corresponding gradiometer cluster over the right hemisphere. To assess the time course of neural memory trace build-up, a 3-way ANOVA was used with factors Stimulus (familiar vs. novel word form), Exposure (initial vs. final sub-blocks), and Sensor (gradiometer pairs), separately for all peaks of interest. Greenhouse-Geisser correction was used (corrected F-values and original degrees of freedom are reported). Effect sizes were assessed using partial eta squared.

Finally, to verify that the participants complied with the task and did not shift their attention to the orthographic stimuli, we assessed their performance on the main non-linguistic target-detection task and measured both the pupil dilation and gaze distance from fixation cross. First, average pupil dilation and distance from fixation were calculated for individual participants only in the time window when the familiar and novel word forms were presented and could be fixated to, separately for initial and final segments of the experiment. To compare whether differences were found in pupil dilation or distance from fixation, a 2-way ANOVA was used with factors Stimulus (familiar vs. novel word form) and Exposure (initial vs. final sub-blocks), separately for pupil dilation and distance from fixation. To assess that possible neural effects were not due to changes in overt attention between beginning and end of exposure, behavioural performance (reaction times, number of misses and false alarms, hit rates, and hit ratios (number of hits/number of targets and false alarms)) was compared between initial and final 1/3 of the experiment using paired t-tests.

Source estimates, computed using both gradiometer and magnetometer data (see, e.g.^30,31^, for discussion on choice of the sensor types to use in source reconstruction analyses), were conducted to pinpoint the neural sources of the effects found on the sensor level data using aforementioned latencies. To limit the risk of type-I errors, source analyses were only conducted if statistically significant main effects or interactions involving Exposure or Stimulus factors were found in the ERF analyses. To assess cortical generators of neural activity underlying such surface MEG dynamics, distributed source reconstruction techniques (weighted minimum-norm current estimates, e.g.^27^) were utilised using Brainstorm software. Default MRI template brain^32^ was used for head model, which was then utilised to create overlapping spheres head models, as suggested by^33^. Noise covariance matrices were determined individually separately for each recording block. The entire recording of the block was used for noise covariance matrix estimation, which was then used to regularise noise covariance in the wMNE analysis. Based on a body of previous studies of neural substrates associated with the language function (see, e.g^34–36^.), source activity waveforms of the mean activity arising from the nodes underlying a priori defined regions of interest (ROIs) located in the temporal lobes (anterior and posterior parts of the superior, middle, and inferior temporal lobes) and inferior frontal regions of the left hemisphere were extracted and analysed further. In order to avoid “double-dipping”, selection of ROIs was done a priori (rather than defining the ROIs post-hoc based on activation landscapes). Ad hoc, neural activity (i.e., cumulative dipole strength over ROI) was also extracted from the bilateral region comprising primary visual (V1) and both left and right fusiform regions, in order to assess if any activity differences could be due to basic visual processing. Similarly to the signal-space statistics, the extracted neural source activity was statistically assessed using a 2-way ANOVA with factors Stimulus (familiar vs. novel word forms) and Exposure (initial vs. final sub-blocks) in each region of interest. Greenhouse-Geisser correction was used (corrected F-values and original degrees of freedom are reported). Effect sizes were assessed using partial eta squared (η^2^). All statistical analyses were conducted using SPSS v. 21 software (IBM Corp, NY, USA). As the exposure-related dynamics of the neural activity was the main focus of the study, a Stimulus * Exposure interaction, and other interactions involving factors Stimulus and Exposure, were of primary interest.

## Results

### Behavioural and eye tracking results

All participants in the experiment were able to complete the behavioural task; the hit rate for targets was 97% (range: 94–100%) and hit ratio 92% (range: 82–98%). The mean reaction time was 717 ms (±64 ms; range: 582–846 ms).

Reaction times improved slightly from the beginning to the end of exposure (732 vs. 704 ms; t(16) = 2.879, p = 0.011) as did hit ratios (74 vs. 87%; t(16) = 2.495, p = 0.024), presumably reflecting online training in detecting non-linguistic targets. Differences between other behavioural measures (hit rate, number of misses and false alarms) did not reach statistical significance.

Analysis of eye-tracking data showed no main effects of Stimulus (pupil dilation F(1,16) = 0.888, p = 0.360, η^2^ = 0.053; distance from fixation F(1,16) = 0.002, p = 0.963, η^2^ < 0.001), neither those of Exposure (pupil dilation F(1,16) = 2.285, p = 0.150, η^2^ = 0.125; distance from fixation F(1,16) = 4.489, p = 0.050, η^2^ = 0.219), nor any interactions between Stimulus and Exposure (pupil dilation F(1,16) = 0.218, p = 0.647, η^2^ = 0.013; distance from fixation F(1,16) = 1.194, p = 0.291, η^2^ = 0.069) were found.

To summarise, no behavioural results corresponding to our effect of interest (interaction between Stimulus and Exposure) were found.

### MEG results

The visual assessment of the GFP waveform from all frontal and temporal gradiometer pairs indicated an initial peak at 98 ms from stimulus onset (see Fig. 2); additional peaks at 198, 313 and 524 ms from stimulus onset were analysed ad hoc, as stated in the methods section. The results from the analyses on these are described in detail below.

### First ERF peak at 98 ms after stimulus onset

**Figure 3:**
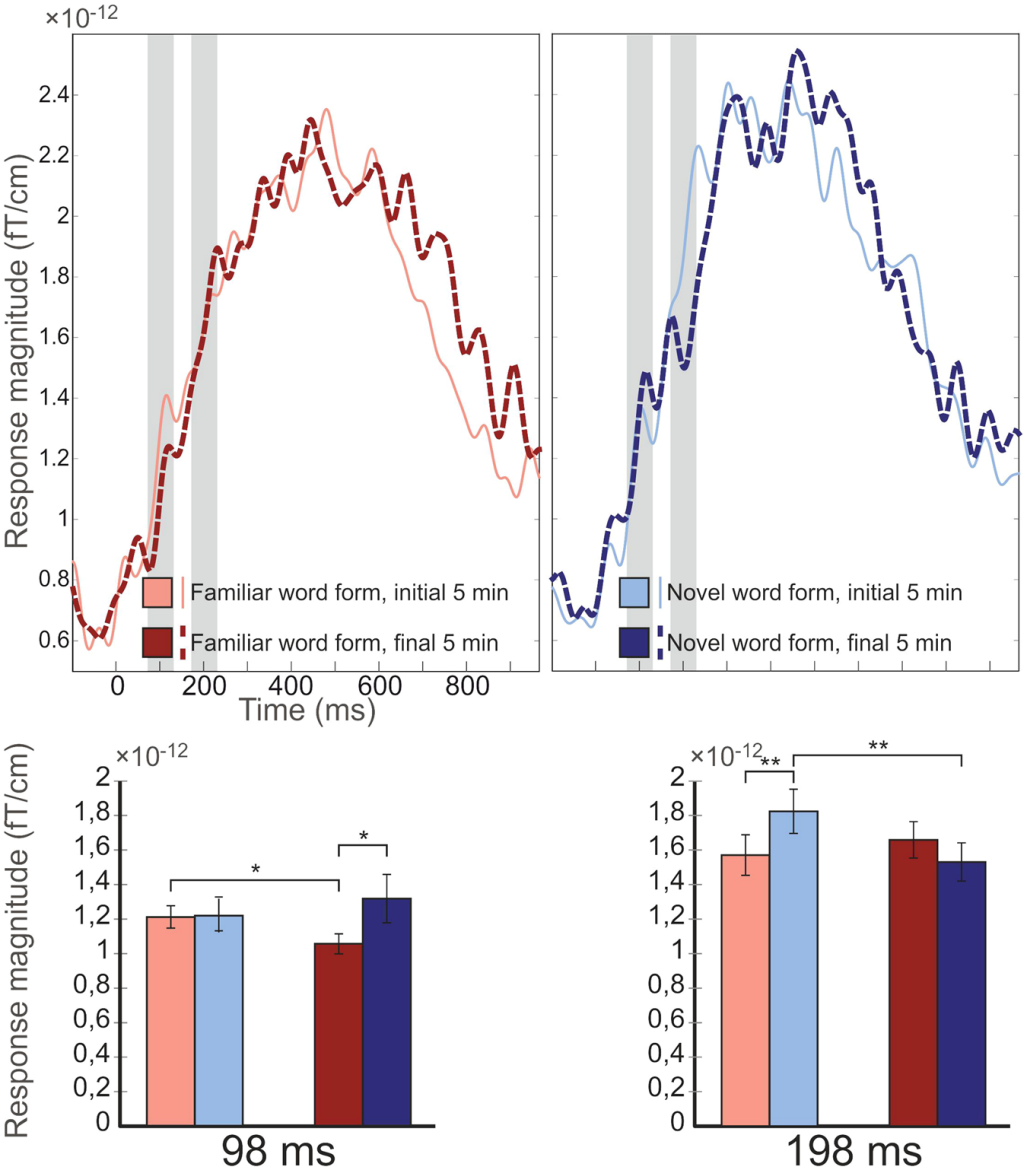
Top: ERF waveforms from the cluster of 14 gradiometers from the left hemisphere for familiar (in red) and novel word forms (in blue) during initial (thin line, lighter colours) and final 33% (thick dashed line, darker colours) of the experiment. Middle: ERF amplitudes for familiar and novel word forms during initial and final 33% of the experiment for both 98 ms and 198 ms peaks from stimulus onset. Error bars denote standard errors of the mean. Asterisks denote statistical significances, *p < 0.05; **p < 0.01.

In the ERF analysis of the left hemispheric cluster, an interaction of Stimulus * Exposure (F(1,16) = 4.742, p = 0.045, η^2^ = 0.229) was found. Post-hoc tests indicated that the effect was due to novel word forms eliciting larger responses than familiar word forms towards the end of the exposure (F(1,16) = 5.030, p < 0.039, η^2^ = 0.239), with responses to familiar, but not novel word forms, diminishing during the experiment (F(1,16) = 6.719, p < 0.020, η^2^ = 0.296) (familiar word forms, beginning: 1.210 ± 0.065 fT/cm; novel word forms, beginning: 1.218 ± 0.094; familiar word forms, end: 1.045 ± 0.058; novel word forms, end: 1.316 ± 0.140). See Fig. 3 for ERF waveforms, amplitudes, and topographies. No effects involving factors Stimulus or Exposure were found in the right-hemisphere sensors, which were tested ad hoc (ps > 0.594, η^2^ < 0.018).

**Figure 4:**
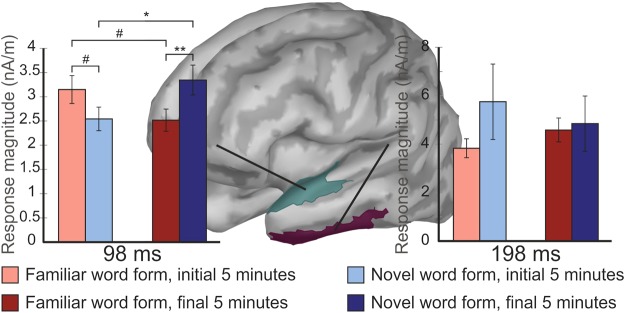
Neural activity in the anterior part of the superior temporal lobe (in cyan) increased for novel word forms (in blue) during from the initial (lighter red) to final 33% (dark red) of the experiment, suggesting formation of new memory traces for novel word forms. Error bars denote standard errors of the mean. Asterisks denote statistical significances. ^#^p < 0.1; *p < 0.05; **p < 0.01.

Analysing source activity, a Stimulus * Exposure interaction (F(9,114) = 2.603, p = 0.027, η^2^ = 0.140) was found in the anterior part of the left superior temporal region (F(1, 16) = 4.522, p = 0.049, η^2^ = 0.221), caused by the activity for novel word forms being larger than that for familiar word forms in the final part of the experiment (familiar word forms, beginning: 3.071 ± 0.310 nA/m; novel word forms, beginning: 2.664 ± 0.367; familiar word forms, end: 2.528 ± 0.262; novel word forms, end: 3.260 ± 0.432; see Fig. 4). Assessment of source activity in V1 revealed only an non-specific effect of Exposure, due to overall activity diminishing during the course of the experiment for all stimuli (F(1,16) = 4.916, p < 0.041, η^2^ = 0.235) but no stimulus specific effects or interactions. No neural effects involving factors Stimulus or Exposure were found in the left fusiform region.

### Subsequent ERF peaks at 198, 313, and 514 ms after stimulus onset

For the subsequent ERF peaks analysed ad hoc (following up on the visual inspection of the ERFs), the analyses indicated that, for the peak at 198 ms, the Stimulus * Exposure interaction was statistically significant over the left hemisphere (F(1,16) = 12.027, p < 0.003, η^2^ = 0.429). Analysing this further, post-hoc tests showed that the effect was due to the response to novel word forms diminishing from the beginning to the end of the experiment (F(1,16) = 15.558, p = 0.001, η^2^ = 0.493). Furthermore, familiar word forms were found to elicit smaller responses than novel word forms during the beginning of the experiment (F(1,16) = 10.246, p < 0.006, η^2^ = 0.390; familiar word forms, beginning: 1.571 ± 0.118 fT/cm; novel word forms, beginning: 1.925 ± 0.128; familiar word forms, end: 1.659 ± 0.105; novel word forms, end: 1.531 ± 0.111; see Fig. 4). No effects involving factors Stimulus or Exposure were found in the right hemisphere (p’s > 0.225, η^2^ < 0.091).

Investigating the neural sources associated with the left-hemispheric effects seen in ERFs at 198 ms after stimulus onset, a near-significant Stimulus * Exposure interaction (F(1,16) = 3.649, p < 0.074, η^2^ = 0.186) was found, originating from the anterior part of the inferior temporal region (familiar word forms, beginning 3.813 ± 0.390 fT/cm; novel word forms, beginning: 5.733 ± 1.556; familiar word forms, end: 4.567 ± 0.493; novel word forms, end: 4.836 ± 1.141; see Fig. 4). No effects or interactions involving Stimulus or Exposure factors were found in the V1 or the left fusiform region (see Supplementary Data).

For the peaks at 313 and 514 ms from stimulus onset, no statistically significant results involving factors Exposure or Stimulus were found in the ERF analyses.

## Discussion

The present study was aimed at investigating putative neural dynamics underpinning automatic acquisition of novel word forms, previously studied in auditory modality, in the visual domain. We adopted an approach developed by the earlier auditory investigations, which presented previously unknown novel word-forms (‘pseudo-words’) repetitively among other filler items, and used similar but familiar word forms as control stimuli. This design, which, by presenting the stimuli outside the focus of attention, is also geared towards testing automatic early stage of language processing in the brain, was here adapted to the visual modality. Specifically, the subjects were occupied by a continuous non-linguistic visual tracking task, while familiar and novel word forms were briefly flashed on the visual field periphery. Our results show an increase of early (~100 ms) neural activity elicited by unattended novel word forms over the course of the short (~15 min) repetitive exposure to these novel items. Furthermore, exposure-related neural effects were also present in later cortical dynamics approximately 200 ms from the stimulus onset, manifest as a decrease of neuromagnetic responses for the novel word forms during the experimental session. These changes in early and late neural dynamics, and the neural sources underpinning them, are discussed in more detail below.

The early neural dynamics, peaking ~100 ms after stimulus onset, demonstrated an enhanced neural activation to novel word forms as a result of short repetitive exposure to these items. This finding appears to be highly similar to the previous reports in the auditory modality, as reviewed in the introduction. Specifically, previous studies have reported rapid increase of neural responsiveness to novel spoken word forms in the 50–120 ms time range from the recognition point of spoken word-forms. What could be the reason for this increase? Typically, repetitive presentation of a stimulus leads to a reduction of brain responses, which is linked to low-level physiological mechanisms such as neuronal refractoriness/sensory habituation^37^. It is also related to higher-level repetition suppression phenomena underpinned by priming, when representational networks are already preactivated and thus their repeated stimulation does not lead to further activity^38^. Such suppression is observed in our study for familiar word forms only, replicating previous EEG studies of the early neural dynamics of word learning. Considering the high degree of similarity between the familiar and novel word forms (which differed by one letter only and comprised psycholinguistically legal combinations) and the identical mode of presentation, it is unlikely that the differences between the familiar and novel word form dynamics could simply be due to low-level visual features. This is further supported by the analysis of V1 activation that indicated similar dynamics of activity in basic visual-processing areas for all stimuli. The differential dynamics may therefore be due to acquisition-related effects of repetitive exposure to novel word forms, where the functional formation and strengthening of a new memory trace leads to an increase in the activity of the newly formed neural memory trace when the respective stimulus is presented at the input. Specifically, it could be argued that the increase of activation seen here is indicative of Hebbian synaptic strengthening resulting from coordinated neuronal activity^21,22^, and appears to be underpinned by sources in the left anterior superior temporal cortical regions that form a part of processing “what” stream^39^. As discussed, such an account has already been previously proposed for auditory word form acquisition in conditions where the word forms are presented outside the focus of attention. This build-up is not possible for already familiar word forms, which may, however, be still subject to repetition-related response suppression. The latter is manifest as amplitude reduction, as indeed observed here and in previous studies^38^.

Such an explanation of this phenomenon is further supported by the topography of this effect, which was seen only in the sensors over the language-dominant left hemisphere, and was localised to parts of the core language system in the temporal lobe, rather than the primary visual cortex.

Thus, the current result indicates that the changes in early neural dynamics associated with exposure-based non-attentive online word form acquisition are rather similar for both visual and auditory modalities. In comparison to previous studies on the neural basis of novel word form acquisition, the current study utilised only visual, not spoken word forms. This is a highly relevant deviation from previous research, as this manipulation allows us to reveal the neural dynamics unaffected by the basic auditory processing that in itself leads to temporal-cortex activity, possibly confounding purely linguistic effects. In sum, our findings suggest that highly similar cortical dynamics are involved in lexicalisation of novel word forms, and points towards a system involved in word form acquisition that is at least partially shared between different modalities. This tentative interpretation certainly needs to be verified in a future experiment utilising a similar paradigm in both auditory and visual modalities in the same subjects.

Finally, in absence of behavioural learning outcomes in the present study, it is reasonable to ask whether this dynamics genuinely reflects the word form acquisition. Thus far only two studies have assessed associations between the word form exposure-related changes in neural dynamics and behavioural learning outcomes, and they report similar dynamics as shown here within the left perisylvian network^9,19^. On basis of similar changes in dynamics in the perisylvian network, although not conclusively, we suggest that the most plausible explanation for it is formation of new memory traces for novel word forms. In line with the present results, studies using somewhat similar methodology and presenting partial word forms visually, have reported distinct changes in neural activity associated with forming a conscious perception of a word form. Specifically, the studies suggest that development of a conscious perception for visually presented word forms is seen as reduced power in the oscillatory activity in the alpha band in the left temporo-parietal regions^40,41^. Future studies using this type of paradigms could potentially investigate the interplay of possibly attention-independent formation of memory traces for word forms and the formation of conscious perception of the presented word form, using ERF analyses, oscillatory brain activity, and assessment of behavioural outcomes.

In addition to the initial component indicative of rapid memory-trace build-up which was at the focus of our investigation, changes in later neural dynamics were also observed approximately 200 ms from stimulus onset. Specifically, these changes in cortical dynamics seem to result from initially stronger activation for novel than familiar word forms diminishing over the course of the experiment. A similar effect was found by Kimppa and others^9^, whose results are indicative of a neural reactivity for novel spoken word forms diminishing in near-identical time range as in the present study. Although the role of the longer-latency component diminishing over the course of the experiment cannot be fully explained on the basis of the current results, it seems plausible that, in the initial stages of the exposure, this component might reflect unsuccessful processes of lexical search as it was apparent for novel word forms only. For instance, an unfamiliar word form may lead to an increased effort of searching for a suitable entry in the mental lexicon and activate a larger neighbourhood/cohort of word forms bearing resemblance to it (see, e.g.^42,43^), contributing to the increased activity, higher than that for familiar word forms whose representations can be easily found earlier on. Such an explanation would predict that, upon successful memory trace formation, this dynamic would subside over the course of exposure, which is exactly what we observe here. In other words, as the novel word form becomes lexicalised, lexical search processes involving it become faster and less intense, diminishing the late neural activity, which follows the earlier peak related to memory trace activation per se. Such a response decrement in a later time range has been reported in EEG studies that focussed on scrutinising N400 dynamics during learning of novel items (see, e.g.^44,45^). A similar pattern is also seen in a reduction of slow BOLD activity known to accompany new word and word-form acquisition in fMRI studies (see, e.g.^43,46,47^). Whereas the current effects, reminiscent of the aforementioned N400 dynamics, commence already at 200 ms, this matches well with a number of previous studies which identified N400-like ERP differences in the same early time range (e.g.^48,49^), provided the stimulus features and presentation timings are exactly controlled, as it was also done here. Thus, we could conclude that the later dynamics may reflect secondary (and possibly top-down controlled) stages in lexical access, which may follow the earlier stage of initial lexical representation activation and depend on the availability of such representations and their successful build-up, serving as an additional index of neural word form acquisition.

The changes in later neural dynamics, seen in sensor space as a decrease of activation for novel word forms over the course of the experiment, were localised to the anterior part of the left inferior temporal gyrus, suggested to play a role in visual word recognition (e.g.^50^). Although the effect was only marginally significant in source space, the observed effect size was medium, suggesting a genuine effect. Hence, this minor divergence between the signal and sensor space results is likely due to predefined ROI used in the analyses. Therefore, it seems highly plausible that initially an unfamiliar word form activates a larger subset of items in the brains’ mental lexicon, the activity which subsides as a novel word form becomes more lexicalised. It thus seems reasonable to assume that the unfamiliar or incongruent word form with native phonology requires the brain to allocate more resources to matching the input onto a known or congruent memory trace, resulting in a neural response of greater magnitude in the left inferior temporal region until a new neural memory trace for it has been formed.

Notably, the current study utilises only a small set of word forms, as the paradigm has been developed in close accordance to the original study conducted in the auditory domain. While this seems reasonable for producing data comparable to the original studies, such a small number of token nevertheless may be insufficient to generalise to all word form acquisition processes (although note that this is generally in line with a number of 10–20 word acquired *per week* during language acquisition^2^). Thus, future studies could use more word forms (e.g.^9,10^) and potentially assess behavioural learning outcomes as well (e.g.^9,19^). The use of learning outcomes may be extremely relevant as our findings indicate that some effects in behavioural performance or eye movements may occur over the duration of an MEG experiment. Assessing how well individuals acquire the novel word forms and whether some background factors (e.g. reading proficiency, general language skills, number of languages acquired, etc.) is especially important to both verify that the observed results are genuinely indicative of general lexical processes and provide more nuanced information on how word form acquisition is associated with other cognitive processes. In addition, it is relevant to assess to what extent the observed changes in neural activity in the current study are indicative of general lexical abilities. An altogether separate avenue for similar future studies could be the acquisition of *meaning* of the novel word forms, which may require different paradigms (such as word-picture association).

Finally, it needs to be noted that several peaks of interest were analysed in the present study, which may increase the risk for type-I error. However, as the results from rapid learning phenomenon are still accumulating, it is relevant to assess all facets of the phenomenon. Over the course of several studies, neural responses most prominently associated with rapid learning seem to be those with latencies approximately within ~100 ms of the uniqueness point. Hence, our findings concerning the second response at ~200 ms from uniqueness point need to be cautiously interpreted for the time being.

To conclude, the present study documents the time-course and online functional changes in the neural activity which may underlie rapid acquisition of novel word forms outside the focus of attention in the visual domain. The enhancement of the activity, which could be interpreted as reflecting a build-up of memory circuits, is seen ~100 ms after the visual word form onset and takes place when the stimuli are not specifically attended to and no stimulus-related or linguistic task is given. This indicates of a rapid and attention-invariant mechanism. This mechanism may benefit from the brains’ pre-existing perception-articulation and phoneme-grapheme correspondence links, specifically in the temporal parts of the left perisylvian network. Both the time course of the neural activity pertaining to acquisition of novel word forms and the underlying sources found in our study are comparable with the findings of previous studies conducted in the auditory modality. Hence, combined with previous literature, the results appear to suggest that the neural mechanism that underpins the formation of neural memory traces of novel linguistic information might be shared by auditory and visual modalities. In sum, the findings presented herein illustrate the fascinating and flexible ability of the human brain to utilise a modality-independent neural mechanism for building new linguistic memory traces.

## Electronic supplementary material

Supplementary material

## Data Availability

The datasets generated during the current study are available from the Cognition and Brain Sciences Unit, Cambridge, UK, on reasonable request to the corresponding author.
