# Supplementary material for: Formation of neocortical memory circuits for unattended written word forms: neuromagnetic evidence
Source: Sci Rep. 2018 Oct 25;8:15829. doi: 10.1038/s41598-018-34029-y (PMC6202413; doi:10.1038/s41598-018-34029-y)
Supplement: Supplementary file 1 — Supplementary material [file 41598_2018_34029_MOESM1_ESM.docx]

Title page

Supplementary material

**Formation of neocortical memory circuits for unattended written word forms: neuromagnetic evidence**

Eino J. Partanen^a,b,*^, Alina Leminen^a,b^, Clare Cook^d^ and Yury Shtyrov^a,c,d^

**Analysis of ERF components between 100 and 200 ms**

As the GFP waveform includes a possible response between 100 and 200 ms, this response, too, was analyzed post hoc for the sake of completeness. Specifically, the peak latency of this possible component is 146 ms after stimulus onset. Using a similar analysis as with other components, a main effect of Stimulus was found: at this latency, novel word forms elicit larger responses than familiar word forms (F(1,16)=5.109, 0=0.038, η2=0.242). No effects related to the study hypotheses were found (Time * Stimulus interaction, p=0.539).

**Analysis of standard responses**

We also assessed, ad hoc, whether similar changes occur in frequently presented word forms as those hypothesized and identified in the infrequently presented ones. As the infrequent stimuli were presented a total of 33 times in the first and final 1/3 of the experiment, responses to a comparable number of frequently presented stimuli are plotted in Figure 1.

**
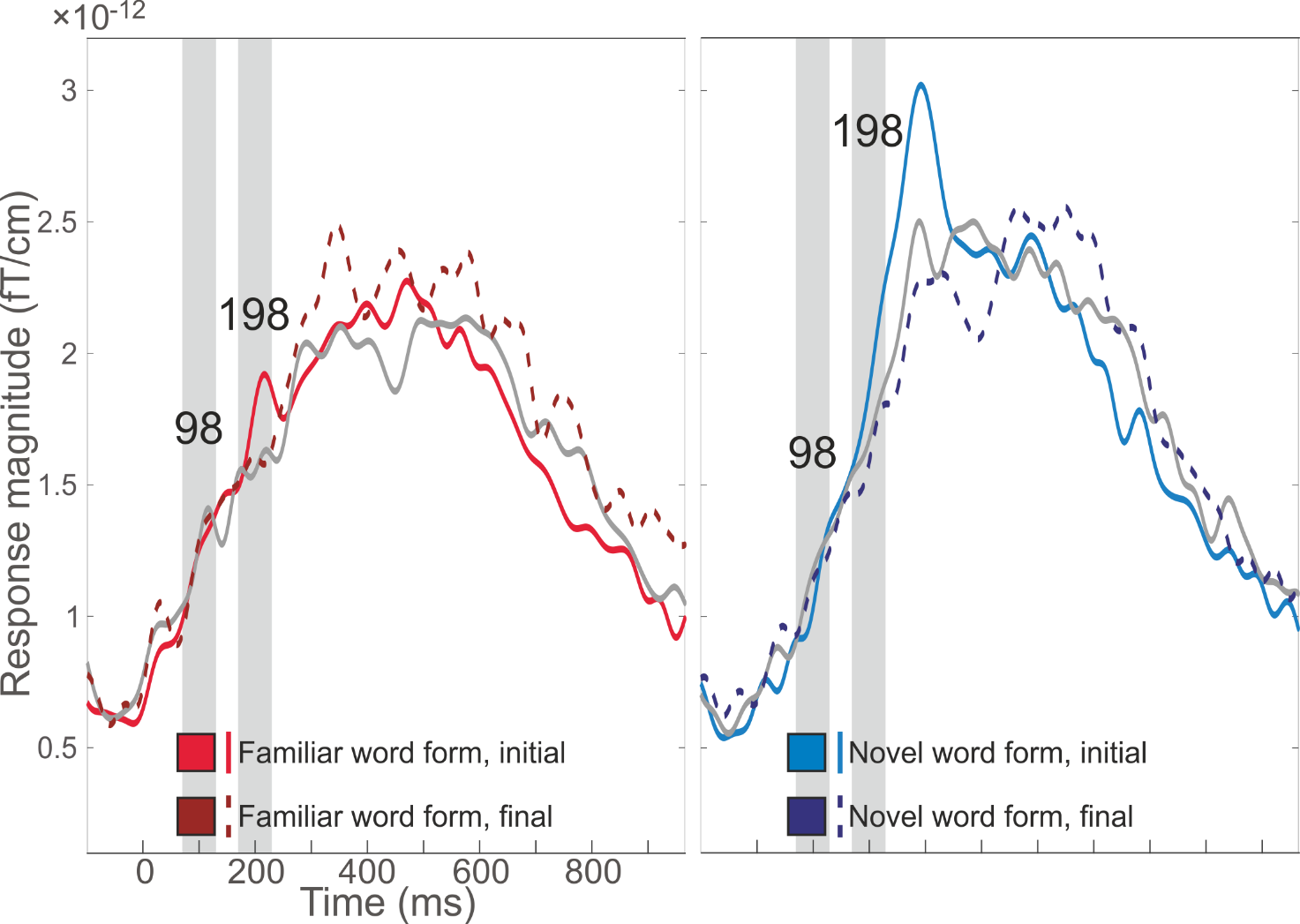
**

**Figure 1.** Analysis of frequently presented stimuli. For novel word forms, an increase of response amplitude at 98 ms from stimulus onset was not observed over the course of the experiment.

*First ERF peak at 98 ms after stimulus onset*

In the ERF analysis of the left hemispheric cluster, no statistically significant Stimulus * Exposure effect was found (F(1,16)=0.015, p=0.905, , η^2^=0.001). However, a statistically significant effect of Stimulus was found, with novel items eliciting larger responses then familiar items (F(1,16)=5.549, p=0.032, η^2^=0.258; novel word forms: 1.212 ± 0.065; familiar word forms: 1.037 ± 0.080).

*Second ERF peak at 198 ms after stimulus onset*

The ERF analyses indicated that neither the Stimulus * Exposure interaction (F(1,16)=0.014, p=0.907, η^2^=0.001) or the main effect of Stimulus (F(1,16)=0.064, p=0.804, , η^2^=0.004) were statistically significant over the left hemisphere sensors.

**Primary visual cortex activation**

The activation of the V1 in time windows of interest (60 ms wide windows centered at ERP peaks at 98 and 198 ms after stimulus onset) are depicted in Figure 2. No statistically significant effects of factor Stimulus, Exposure, nor a statistically significant interaction of Stimulus and Exposure was found.


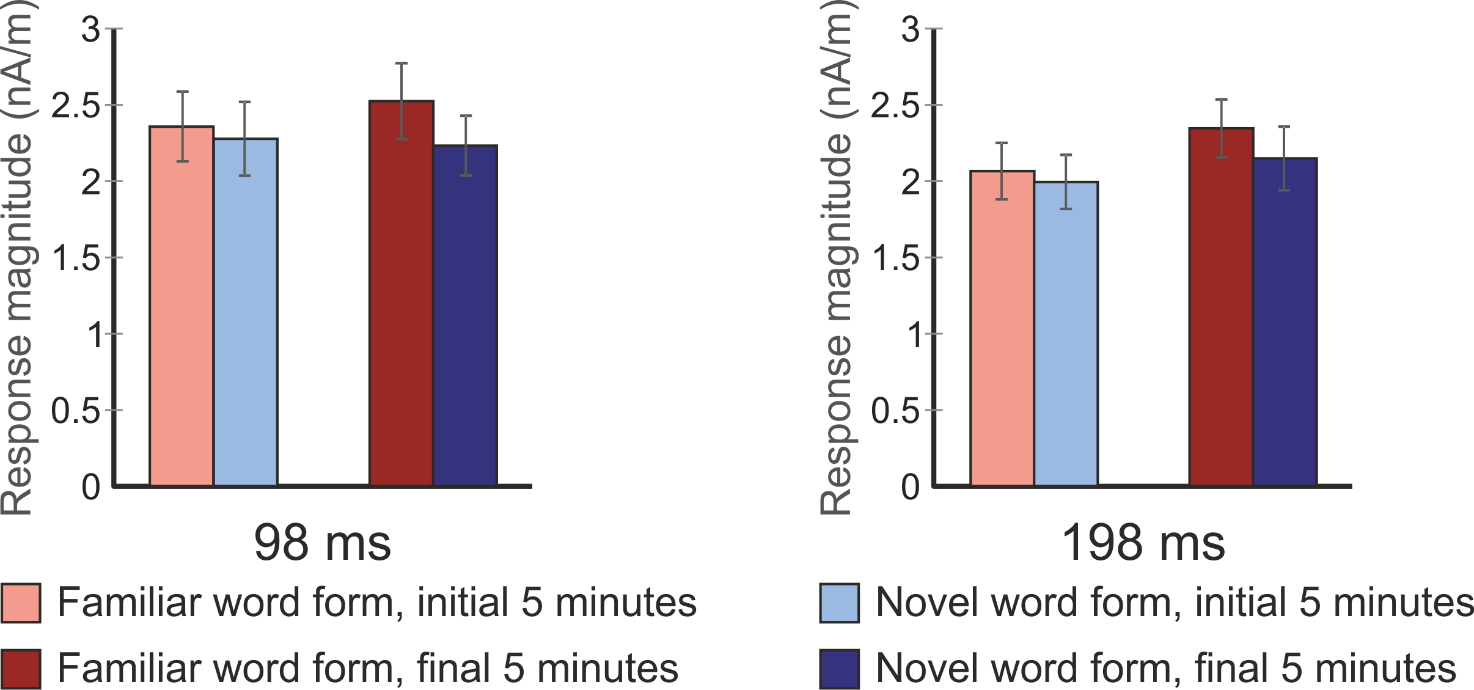


**Figure 2.** Primary visual cortex activation during the experiment. The left bar graph denotes neural activity originating from the V1 region at 98 ms after stimulus onset while the bar graph on the right illustrates the activity at 198 ms after stimulus onset.

**Distributed source models**

The distributed source models of contrasts of interest (Stimulus, Exposure, and interaction of Stimulus and Exposure) are depicted in Figure 3 and Figure 4. The activation depicted in the Figures 3 and 4 illustrate the average neural activity in the time window of interest, with identical time windows from which the mean ERF activity is calculated from (e.g., 68-128ms and 168-228ms).


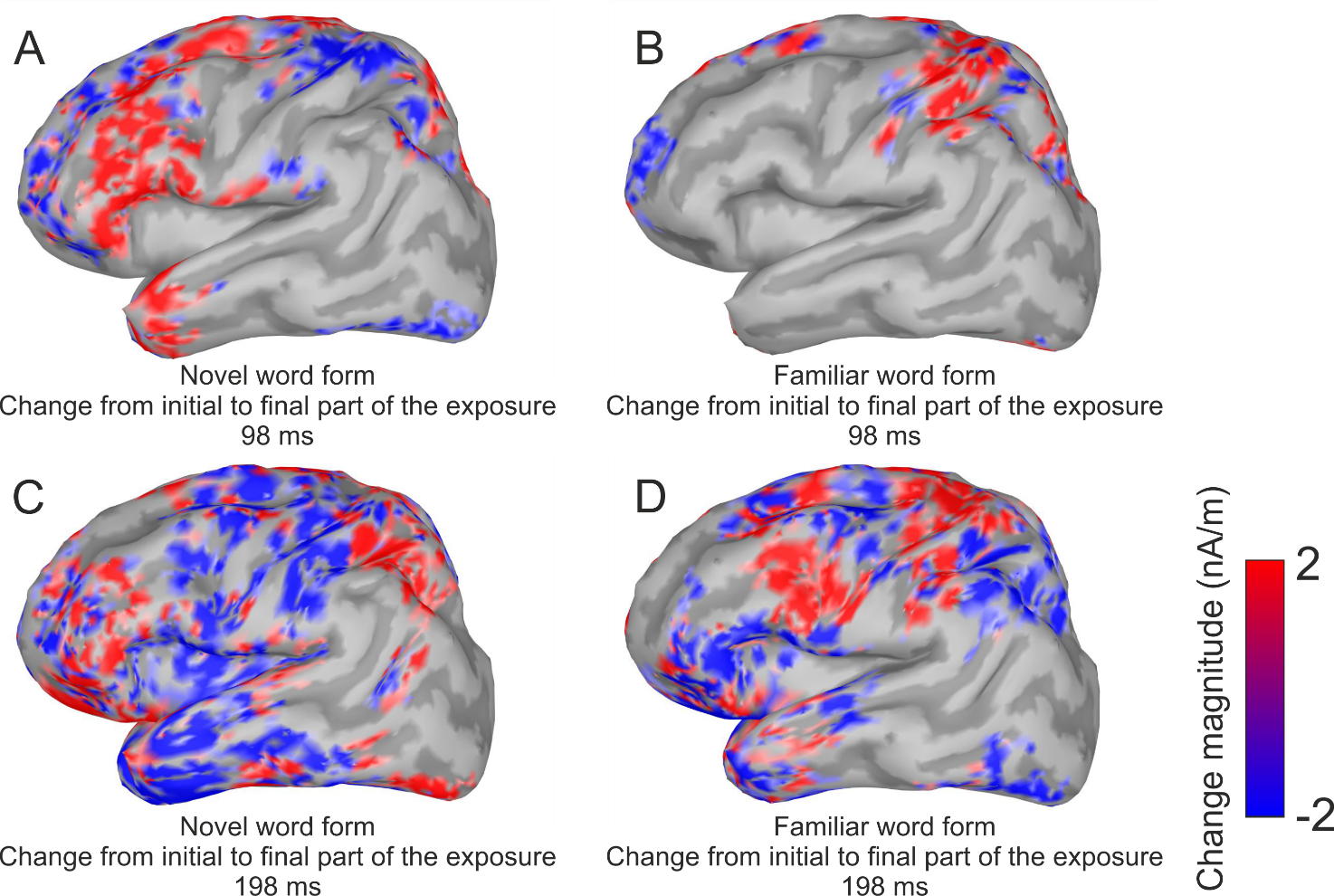


**Figure 3.** The changes in neural activity between beginning and end of the experiment. Increase in activation from beginning to the end of the experiment are shown in red while decrease in activation is depicted in blue.


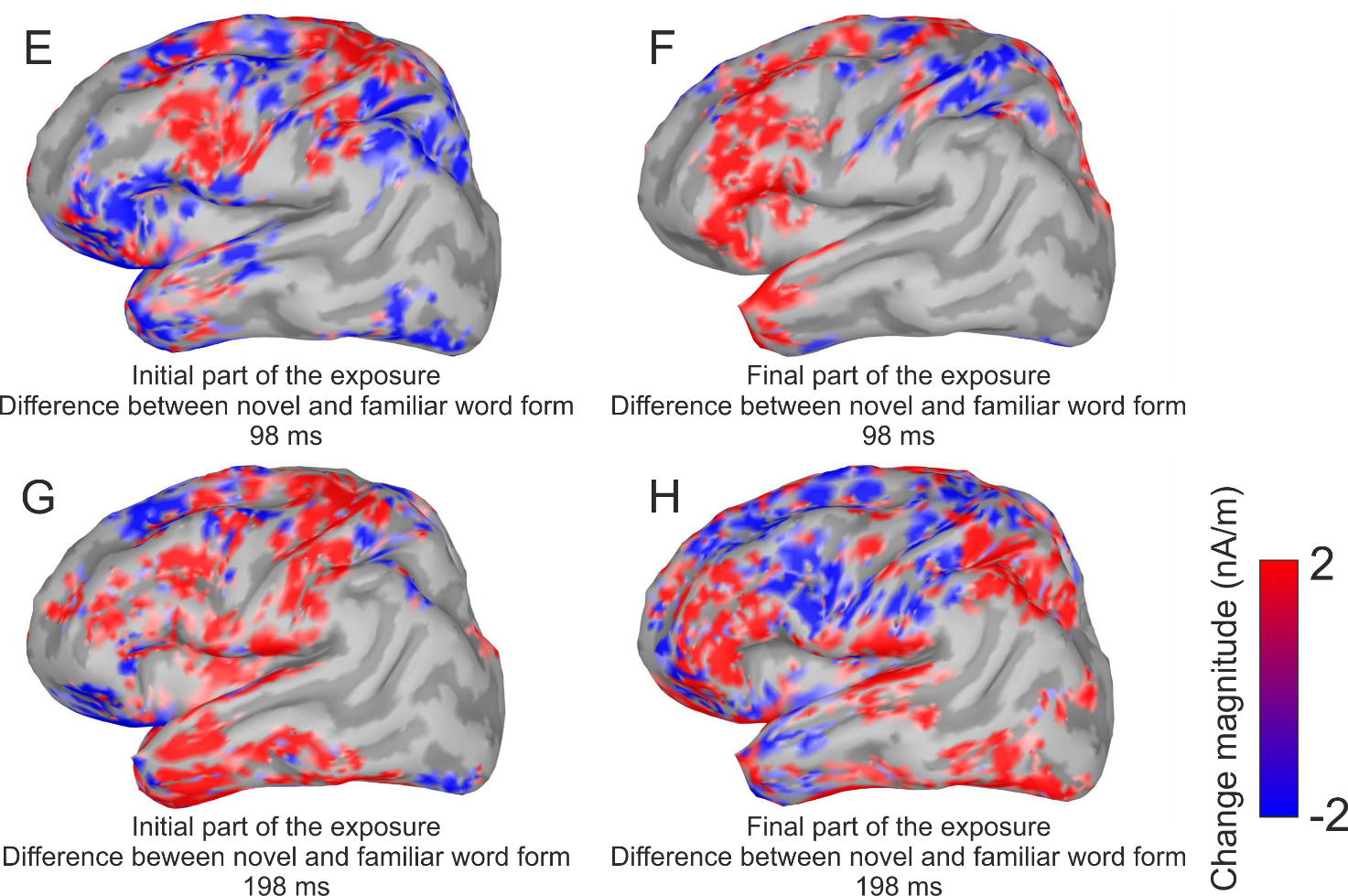


**Figure 4.** Differences in neural activity between novel and familiar word forms in both beginning and end of the experiment. Larger activity for novel than familiar word forms is show in red while larger activity for familiar than novel word forms is shown in blue.
